# Long noncoding RNA Gm31629 protects against mucosal damage in experimental colitis via YB-1/E2F pathway

**DOI:** 10.1172/jci.insight.150091

**Published:** 2022-03-22

**Authors:** Xu Feng, Ye Xiao, Jian He, Mi Yang, Qi Guo, Tian Su, Yan Huang, Jun Yi, Chang-Jun Li, Xiang-Hang Luo, Xiao-Wei Liu, Hai-Yan Zhou

**Affiliations:** 1 Department of Endocrinology, Endocrinology Research Center,; 2 Department of Gastroenterology, and; 3 National Clinical Research Center for Geriatric Disorders, Xiangya Hospital of Central South University, Changsha, Hunan, China.

**Keywords:** Gastroenterology, Therapeutics, Cell cycle, Inflammatory bowel disease

## Abstract

Mucosal healing is a key treatment goal for inflammatory bowel disease, and adequate epithelial regeneration is required for an intact gut epithelium. However, the underlying mechanism for mucosal healing is unclear. Long noncoding RNAs (lncRNAs) have been reported to be involved in the development of inflammatory bowel disease. Here, we report that a lncRNA named Gm31629 decreased in intestinal epithelial cells in response to inflammatory stimulation. Gm31629 deficiency led to exacerbated intestinal inflammation and delayed epithelial regeneration in dextran sulfate sodium–induced (DSS-induced) colitis model. Mechanistically, Gm31629 promoted E2F pathways and cell proliferation by stabilizing Y-box protein 1 (YB-1), thus facilitating epithelial regeneration. Genetic overexpression of Gm31629 protected against DSS-induced colitis in vivo. Theaflavin 3-gallate, a natural compound mimicking Gm31629, alleviated DSS-induced epithelial inflammation and mucosal damage. These results demonstrate an essential role of lncRNA Gm31629 in linking intestinal inflammation and epithelial cell proliferation, providing a potential therapeutic approach to inflammatory bowel disease.

## Introduction

Ulcerative colitis is a form of inflammatory bowel disease characterized by relapsing and remitting diarrhea and bloody stools (1, 2). Impaired intestinal epithelial barrier and restricted mucosal inflammation ranging from rectum to proximal colon characterized the common feature of ulcerative colitis pathology (2, 3). Usually, the development of ulcerative colitis is initially driven by intestinal epithelial dysfunction, which includes increased apoptotic epithelial cells and disrupted tight junctions, leading to epithelial barrier defect and resultant lamina propria inflammation (3–5). Thus, adequate intestinal epithelial regeneration is an essential precondition for mucosal healing and clinical remission (6–8). However, the precise mechanism of epithelial regeneration and inflammation is not clear.

Long noncoding RNAs (lncRNAs) are defined as a class of non-protein-coding transcripts longer than 200 nucleotides, which exert their function by interacting with nucleic acids and proteins (9, 10). Emerging evidence has proven that dysregulated lncRNAs play important roles in the development of various diseases, including inflammatory bowel disease (11–14). In previous studies, lncRNAs have been proven to be involved in the regulation of intestinal epithelial cell apoptosis (15, 16), regeneration (17–19), and inflammatory response (20, 21). We previously identified a lncRNA, named Gm31629, which is abundant in BM mesenchymal cells (BMSCs) and hypothalamic neural stem cells (htNSCs) at early ages of mice (14, 22). Gm31629 was found to regulate cell proliferation and differentiation of BMSCs and htNSCs (14, 22). These findings raise a question whether Gm31629 would play a role in intestinal epithelial regeneration and ulcerative colitis progression.

In this report, we show that colonic Gm31629 expression decreased in dextran sulfate sodium–induced (DSS-induced) colitis and LPS-induced systemic inflammation. Human orthologue Gm31629 also decreased in inflamed colon tissues compared with the unaffected tissues. Gm31629-KO (Gm31629^–/–^) mice exhibited exacerbated inflammation and delayed mucosal healing in DSS-induced colitis. RNA-Seq analysis of colon tissues from Gm31629^–/–^ mice and WT littermates revealed that broadly dysregulated genes were involved in inflammatory response and cell cycle. Mechanistically, Gm31629 promotes E2F pathways and cell proliferation by stabilizing YB-1 protein, thus facilitating epithelial regeneration. Genetic overexpression of Gm31629 by i.p. adenovirus delivery protected against DSS-induced colitis in mice. Theaflavin 3-gallate (TF2A), a small naturally occurring compound, functioned as a mimic of Gm31629 and ameliorated DSS-induced epithelial inflammation and damage. Taken together, our study reveals a critical role of Gm31629 in intestinal homeostasis and may represent a novel therapeutic target against ulcerative colitis.

## Results

### Inflammation leads to reduced intestinal Gm31629 expression.

**Figure 1:**
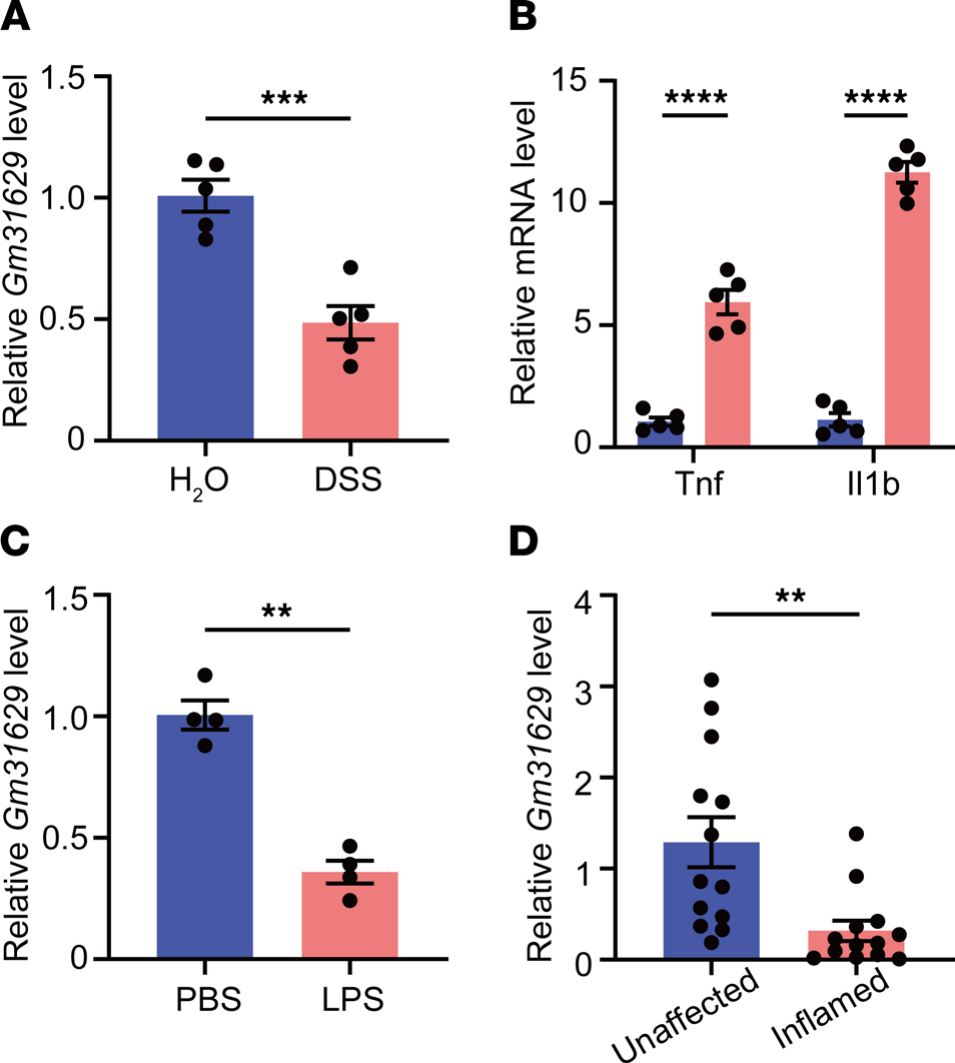
Expression of intestinal Gm31629 decreases in response to inflammatory stimuli. (**A** and **B**) Eight-week-old WT mice were subjected to 3% DSS in drinking water or tap water for 7 days and sacrificed on day 8. qPCR analysis of *Gm31629* (**A**), *Tnf*, and *Il1b* (**B**) in colon tissues of mice (*n* = 5, data represent mean ± SEM, 2-tailed Student’s *t* test). (**C**) Eight-week-old WT mice were i.p. injected with 2 mg/kg LPS or PBS for 24 hours. qPCR analysis of *Gm31629* in colon tissues of mice (*n* = 4, data represent mean ± SEM, 2-tailed Student’s *t* test). (**D**) qPCR analysis of *Gm31629* in colonic biopsies from inflamed and unaffected mucosa of patients with ulcerative colitis (*n* = 13, data represent mean ± SEM, 2-tailed Welch’s *t* test). **P < 0.01; ***P < 0.001; ****P < 0.0001.

To investigate the role of Gm31629 in intestinal homeostasis, we first examined whether intestinal mucosa could express Gm31629. RNA FISH validated the constitutive expression of Gm31629 in intestinal epithelial cells, including that of intestinal stem cells residing at the base of the colon crypt (Supplemental Figure 1A; supplemental material available online with this article; https://doi.org/10.1172/jci.insight.150091DS1). We next employed a DSS-induced intestinal inflammation model that mimicked epithelial damage features in human ulcerative colitis. DSS-treated mice scored high on the disease activity index (DAI); they showed continuous body weight loss, progressive diarrhea, and bloody stools, which were absent in mice treated with tap water (Supplemental Figure 2, A and B). The colon length was shortened in DSS-treated mice, reflecting severity of inflammation (Supplemental Figure 2C). Accordingly, histological analysis displayed apparent epithelial damage and inflammatory cell infiltration in colon tissues of DSS-treated mice (Supplemental Figure 2, D and E). Using quantitative PCR (qPCR) analysis, we found that expression of colonic Gm31629 decreased by about 2-fold, while the expression of *Tnf* and *Il1b* dramatically increased after DSS administration (Figure 1, A and B). We then used a LPS challenge model featuring an acute systemic inflammatory response. I.p. injection of LPS also resulted in reduction of colonic Gm31629 expression (Figure 1C). Considering that Gm31629 showed a fairly high evolutionary conservation, we collected colon biopsy samples from 13 patients with ulcerative colitis. Consistently, human orthologue Gm31629 was downregulated in inflamed tissues compared with the unaffected ones (Figure 1D). These data indicate a role of Gm31629 in intestinal inflammation.

### Loss of Gm31629 sensitizes mice to DSS-induced colitis and delays epithelial regeneration.

**Figure 2:**
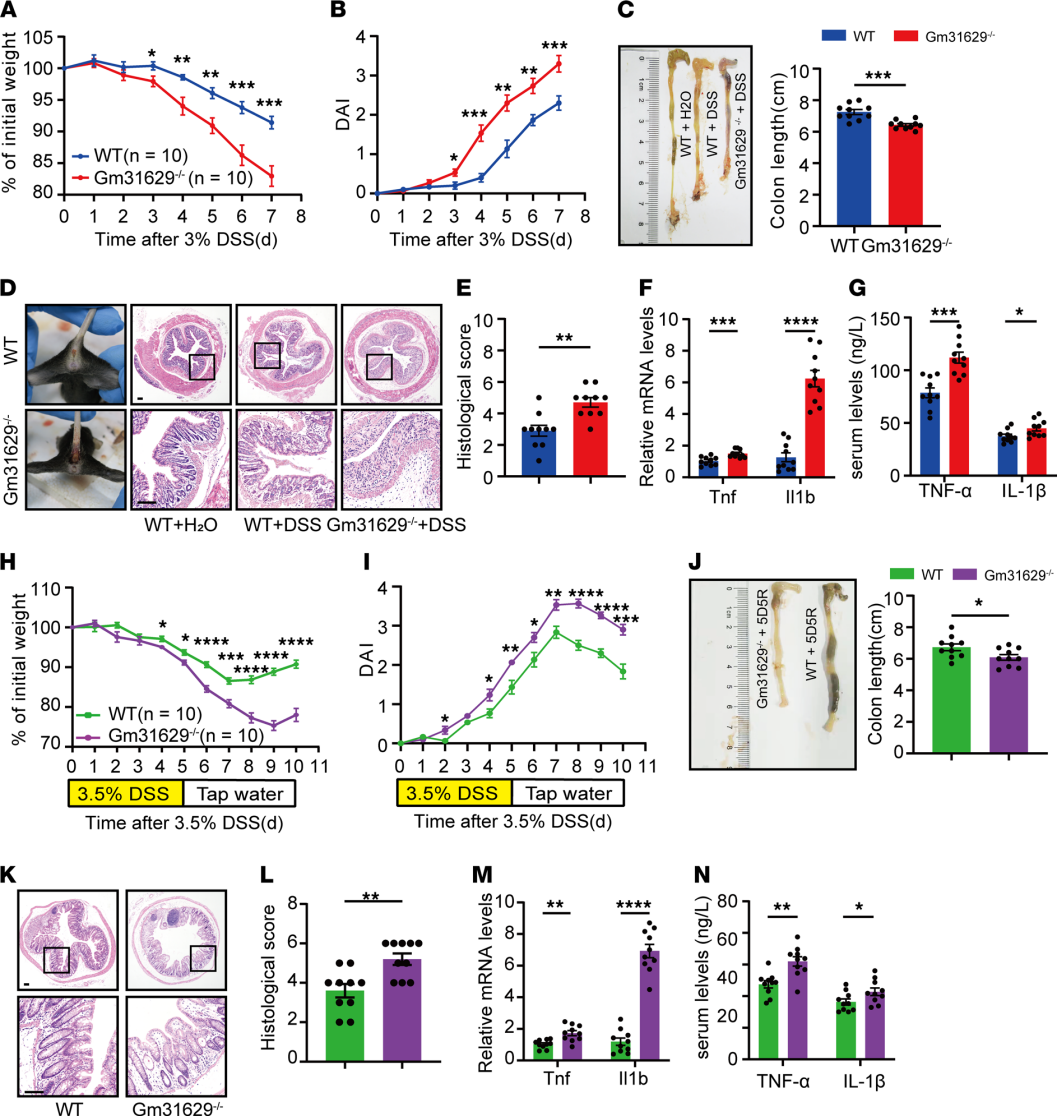
Gm31629 depletion exacerbates DSS-induced colitis and delays intestinal regeneration. (**A**–**G**) Eight-week-old Gm31629^–/–^ mice and littermate WT mice were subjected to 3% DSS in drinking water for 7 days (*n* = 10). (**A**) Body weight changes of mice. (**B**) DAI of mice during the indicated period. (**C**) Gross pictures of colon and measurement of colon length. (**D**) Gross pictures of rectal bleeding and H&E stained section of colon tissues. Scale bar: 100 μm. (**E**) Quantification of histological score from colonic sections in **D**. (**F**) qPCR analysis of *Tnf* and *Il1b* in colon tissues. (**G**) Quantification of serum TNF-α and IL-1β protein level. (**H**–**N**) Eight-week-old Gm31629^–/–^ mice and littermate WT mice were subjected to 3.5% DSS in drinking water for 5 days, followed by 5 days of recovery (*n* = 10). (**H**) Body weight changes of mice. (**I**) DAI of mice. (**J**) Gross pictures of colon and measurement of colon length after 10-day cycle. (**K**) H&E-stained section of colon after 10-day cycle. Scale bar: 100 μm. (**L**) Quantification of histological score from colonic sections in **K**. (**M**) qPCR analysis of *Tnf* and *Il1b* in colon tissues after 10-day cycle. (**N**) Quantification of serum TNF-α and IL-1β protein level after 10-day cycle. All data were represented as mean ± SEM. For comparisons of 2 groups, a 2-tailed Student’s *t* test was performed. *P < 0.05; **P < 0.01; ***P < 0.001; ****P < 0.0001.

In our previous study, Gm31629^–/–^ mice have been well established (Supplemental Figure 1, B and C) (14, 22). These mice have no apparent gross phenotype or histology changes concerning the intestinal tract (Supplemental Figure 1D). Gm31629^–/–^ mice and WT littermates were administered 3% DSS in drinking water for 7 days. Compared with WT mice, Gm31629^–/–^ mice exhibited more weight loss, accompanied by a higher DAI score: more severe diarrhea and rectal bleeding (Figure 2, A and B). By the end of the treatment, the average colon length of Gm31629^–/–^ mice was shorter than that of WT mice (Figure 2C). As for histological evaluation of colon tissue sections, Gm31629^–/–^ mice showed extensive epithelial damage and bulks of inflammatory cell infiltration, and they scored higher for inflammation-associated histological changes than their WT littermates (Figure 2, D and E). qPCR analysis showed higher colonic *Tnf* and *Il1b* mRNA levels in Gm31629^–/–^ mice, which was concordant with increased serum levels of TNF-α and IL-1β, respectively (Figure 2, F and G). These data indicate that Gm31629 depletion aggravated DSS-induced colonic inflammation. Of note, despite continuous intestinal inflammation and epithelial damage, intestinal epithelial cells regenerated throughout DSS treatment. To determine the effect of Gm31629 depletion on epithelial regeneration, Gm31629^–/–^ mice and WT mice were next administered 3.5% DSS for 5 days followed by 5 days of recovery. After a 10-day injury and recovery cycle, Gm31629^–/–^ mice showed imperceptible weight recovery, whereas WT mice started to regain body weight on the third day of the recovery phase (Figure 2H). Similarly, Gm31629^–/–^ mice gained higher DAI with shorter colon length than WT mice (Figure 2, I and J). The histological score of colon tissue sections confirmed weaker repairment of intestinal epithelium in Gm31629^–/–^ mice (Figure 2, K and L). In addition, mRNA levels of colonic *Tnf* and *Il1b* and serum levels of TNF-α and IL-1β in Gm31629^–/–^ mice significantly increased in contrast to WT mice (Figure 2, M and N). Together, the results demonstrate an essential role of Gm31629 in intestinal inflammation and epithelial regeneration.

### RNA-Seq analysis reveals that Gm31629 participates in the cell cycle regulation in colitis.

**Figure 3:**
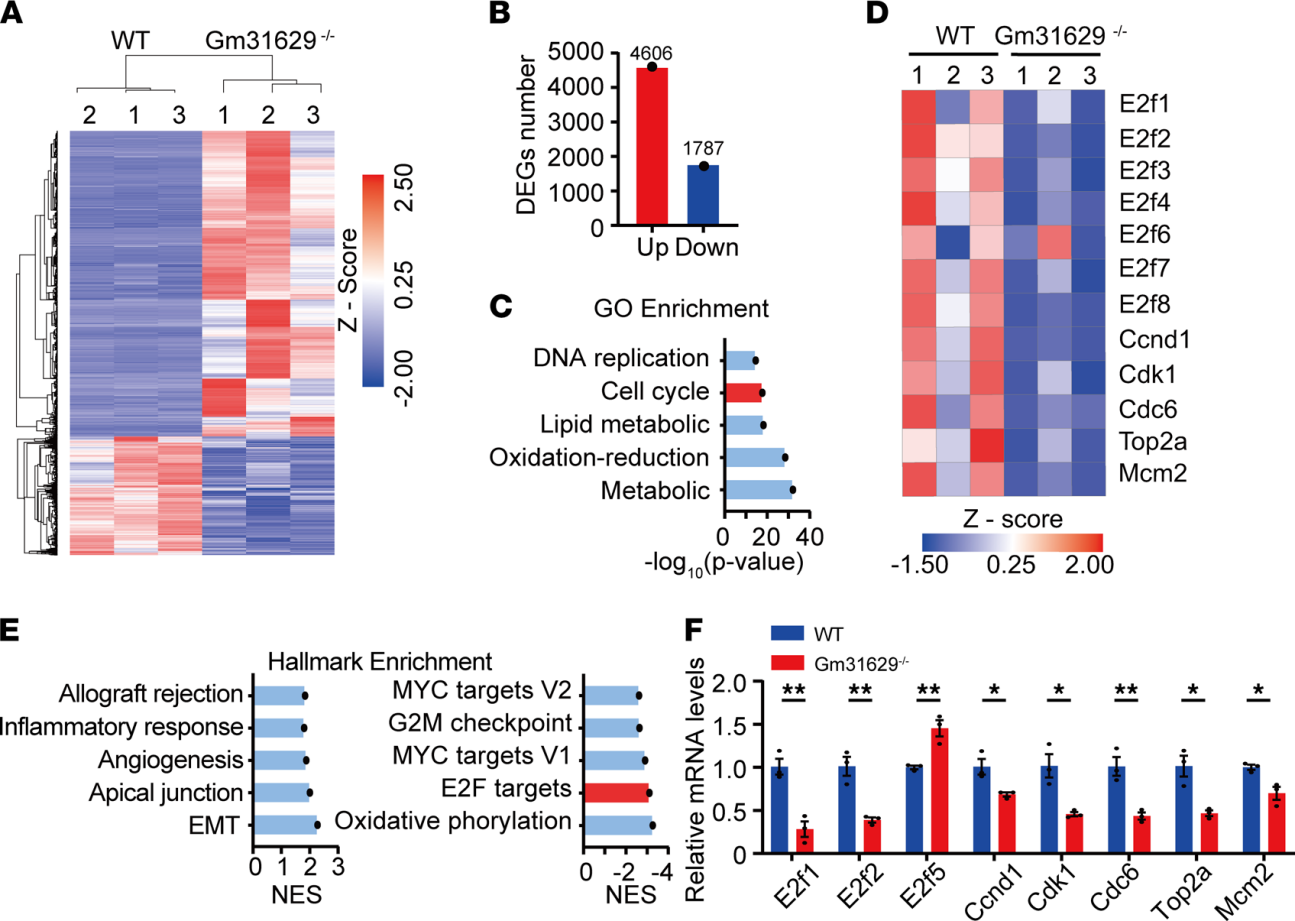
RNA-Seq analysis reveals Gm31629 participates in the cell cycle regulation in colitis. Gm31629^–/–^ mice and littermate WT mice were subjected to 3% DSS for 7 days, and total RNA in colon tissues was isolated for RNA-Seq analysis (*n* = 3). (**A**) Heatmap of differentially expressed genes in colon tissues from Gm31629^–/–^ mice and littermate WT mice (*P* < 0.05 and |log_2_ fold change| > 1). (**B**) Quantification of differentially expressed gene numbers in **A**. (**C**) The top 5 most significantly altered biological processes in GO analysis. (**D**) Heatmap of differentially expressed genes enriched in cell cycle. (**E**) GSEA analysis displayed top 5 pathways upregulated (left) and downregulated (right) hallmark gene sets based on normalized enrichment score (NES) in Gm31629^–/–^ mice versus the littermates. (**F**) qPCR analysis of differentially expressed genes enriched in E2F targets pathway (*n* = 3, data represent mean ± SEM, 2-tailed Student’s *t* test). *P < 0.05; **P < 0.01.

To further evaluate the functional role of Gm31629 in colitis, we isolated total RNA from colon tissues of Gm31629^–/–^ and WT mice for RNA-Seq. The data reveal that 4606 genes were upregulated and 1787 genes were downregulated in colon tissues of Gm31629^–/–^ mice after DSS treatment (Figure 3, A and B). We then performed gene ontology (GO) enrichment of differentially expressed genes to explore biological processes that were affected by Gm31629 KO. The most significantly affected biological processes included metabolic process, oxidation-reduction process, lipid metabolic process, cell cycle, and DNA replication (Figure 3C). Among the most significantly affected processes and differentially expressed genes, we paid our attention to a list of genes associated with cell cycle, including *E2f1*, *E2f2*, *E2f3*, *Ccnd1*, *Cdk1*, *Cdc6*, *Top2a*, and *Mcm2*, which were all downregulated in Gm31629^–/–^ mice (Figure 3D). We further performed gene set enrichment analysis (GSEA) based on hallmark gene sets and demonstrated an enhanced inflammatory response pathway in Gm31629^–/–^ mice, which was consistent with the mouse phenotype (Figure 3E). Downregulated pathways involved oxidative phosphorylation, E2F targets, MYC targets, and G2M checkpoint (Figure 3E). Of note, differentially expressed genes enriched in GO term cell cycle shared considerable overlap with those enriched in E2F targets, which is in accordance with the fact that the E2F family members are the major transcriptional regulators in cell cycle (23–25). The expression changes of target genes were further validated by qPCR (Figure 3F). These results suggest that Gm31629 may regulate cell cycle through E2F pathways, thus promoting epithelial regeneration during DSS treatment.

### Gm31629 deficiency induces cell cycle arrest in the S-phase and inhibits intestinal stem cell proliferation.

**Figure 4:**
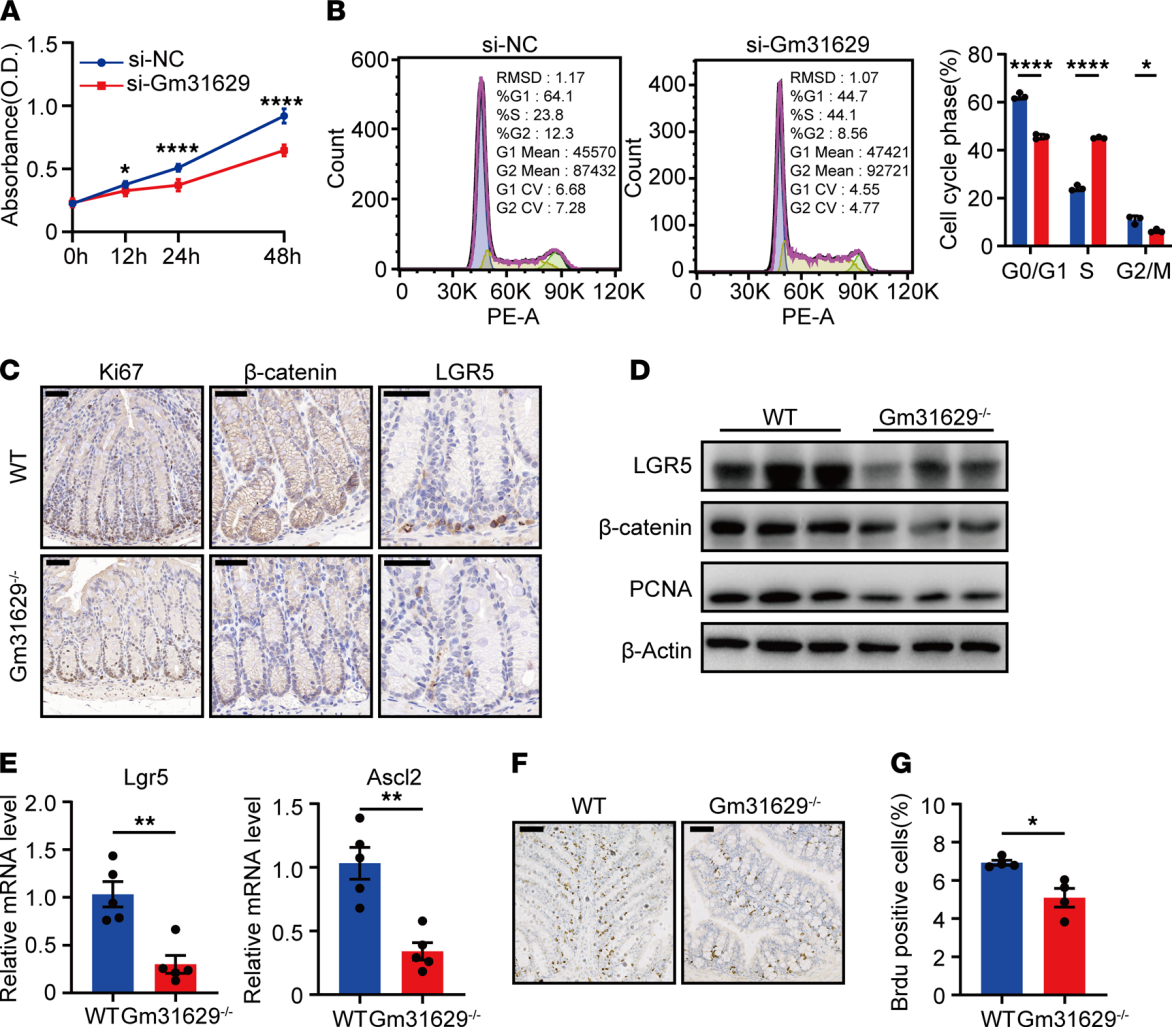
Gm31629 deficiency affects cell cycle and inhibits cell proliferation. (**A**) Cell proliferation assay of CT26.WT cells transfected with si-Gm31629 or control siRNA (*n* = 6). (**B**) Cell cycle analysis of CT26.WT cells transfected with si-Gm31629 or control siRNA (*n* = 3). (**C**) Representative images of Ki-67, β-catenin, and LGR5 staining of colon samples (*n* = 3). Scale bar: 50 μm. (**D**) The protein level of LGR5, β-catenin, and PCNA in colon tissues (*n* = 3). (**E**) qPCR analysis of *Lgr5* and *Ascl2* in colon tissues (*n* = 5). (**F**) Representative images of BrdU staining of colon tissues from LPS-treated Gm31629^–/–^ mice and littermate WT mice (*n* = 4). Scale bar: 100 μm. (**G**) Quantification of BrdU^+^ cells in **F** (*n* = 4). All data were represented as mean ± SEM. For comparisons of 2 groups, a 2-tailed Student’s *t* test was performed. *P < 0.05; **P < 0.01; ****P < 0.0001.

To confirm the role of Gm31629 in cell cycle, cell proliferation assay was performed on CT26.WT colon carcinoma cells transfected with Gm31629 targeted siRNAs and control siRNAs. The results of Cell Counting Kit-8 assay displayed that knockdown of Gm31629 obviously inhibited cell proliferation (Figure 4A). Meanwhile, cell cycle analysis showed a significant increase in S-phase and decreases in G0/G1 and G2/M phase after Gm31629 knockdown (Figure 4B). Suppressing E2F-dependent transcription could limit replication capacity and rates during S-phase and, thus, lengthen S-phase (25). These data suggest that Gm31629 deficiency led to S-phase arrest and inhibited cell proliferation via E2F pathway. Given the constitutive expression of Gm31629 in intestinal epithelial cells, including intestinal stem cells, which reside at the base of the crypt (Supplemental Figure 1A), we speculated that Gm31629 deficiency inhibits intestinal stem cell proliferation and, thus, impairs epithelial regeneration. We next examined intestinal stem cell–related genes in vivo. β-Catenin, the key regulator of intestinal stem cells, significantly decreased in colon tissues of Gm31629^–/–^ mice (Figure 4, C and D). Lgr5 and Ascl2, the markers of intestinal stem cells and the targets of Wnt/β-catenin signaling (26), decreased in colon tissues of Gm31629^–/–^ mice (Figure 4, C–E). Proliferation-related markers, such as PCNA and Ki-67 also markedly decreased in colon tissues of Gm31629^–/–^ mice (Figure 4, C and D). We further conducted BrdU labeling assays in mice after LPS challenge to detect the proliferation activity in vivo. The results showed significantly decreased BrdU^+^ cells in Gm31629^–/–^ mice (Figure 4, F and G), suggesting decreased proliferation of intestinal stem cells. Together, we concluded that Gm31629 deficiency leads to S-phase arrest and inhibits intestinal stem cells proliferation.

### Gm31629 regulates E2F pathways by stabilizing YB-1 protein.

**Figure 5:**
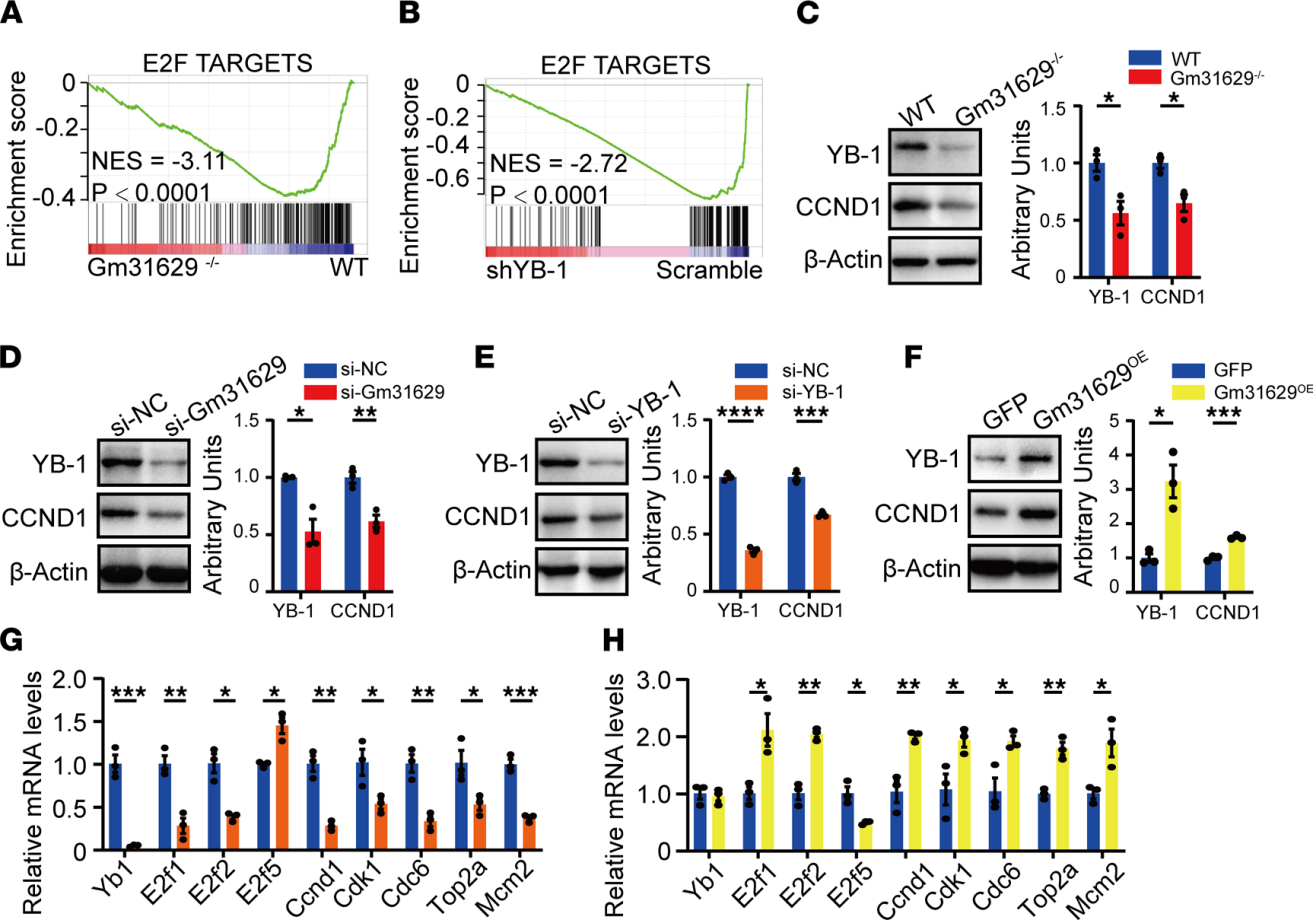
Gm31629 regulates E2F pathways via stabilizing YB-1. (**A**) GSEA plot of E2F targets biological pathway in Gm31629^–/–^ mice versus WT mice (*n* = 3). (**B**) GSEA was performed based on RNA-Seq data from human YB-1 knockdown adipose-derived stem cells samples (*n* = 3), and enriched biological pathway was plotted. (**C**) The protein level of YB-1 and CCND1 in colon tissues from Gm31629^–/–^ mice and littermate WT mice (*n* = 3). (**D**) The protein level of YB-1 and CCND1 in CT26.WT cells transfected with siRNAs targeting Gm31629 and scrambles (*n* = 3). (**E**) The protein level of YB-1 and CCND1 in CT26.WT cells transfected with siRNAs targeting YB-1 and scrambles (*n* = 3). (**F**) The protein level of YB-1 and CCND1 in CT26.WT cells overexpressing Gm31629 (*n* = 3). (**G**) qPCR analysis of *Yb1* and E2F targets genes in CT26.WT cells transfected with siRNAs targeting YB-1 and scrambles (*n* = 3). (**H**) qPCR analysis of *Yb-1* and E2F targets genes in CT26.WT cells overexpressing Gm31629 (*n* = 3). All data were represented as mean ± SEM. For comparisons of 2 groups, a 2-tailed Student’s *t* test was performed. *P < 0.05; **P < 0.01; ***P < 0.001; ****P < 0.0001.

We next questioned how Gm31629 regulates E2F pathway. In our previous study, Gm31629 could bind to YB-1 protein and prevent its degradation, thus, regulating downstream signaling (14). Meanwhile, another study has proven a positive correlation between YB-1 and E2F target genes (23). GSEA using public RNA-Seq data consisting of samples from human adipose–derived stem cells (27) showed that YB-1 knockdown significantly downregulated E2F targets, and the GSEA plot was extremely similar to that of Gm31629^–/–^ mice versus WT mice. These results strongly suggest a role of YB-1 in E2F pathways (Figure 5, A and B). We therefore assumed if Gm31629 could regulate gene expression of E2F pathways by stabilizing YB-1 protein. To validate our hypothesis, we next examined YB-1 expression in vivo and in vitro. YB-1 protein level decreased in colon tissues of Gm31629^–/–^ mice as expected, accompanied by decreased CCND1 protein level (Figure 5C). Decreased YB-1 and CCND1 protein levels were also observed in CT26.WT cells transfected with Gm31629 siRNA (si-Gm31629) (Figure 5D). In addition, Yb-1 knockdown led to downregulated protein level of CCND1 and mRNA levels of E2F target genes (Figure 5, E and G). On the contrary, Gm31629 overexpression increased protein levels of YB-1 and CCND1, as well as mRNA levels of E2F target genes, without changing YB-1 mRNA level (Figure 5, F and H). These results demonstrate that Gm31629 regulates E2F pathways by stabilizing YB-1 protein.

### Overexpression of Gm31629 protects against DSS-induced colitis.

**Figure 6:**
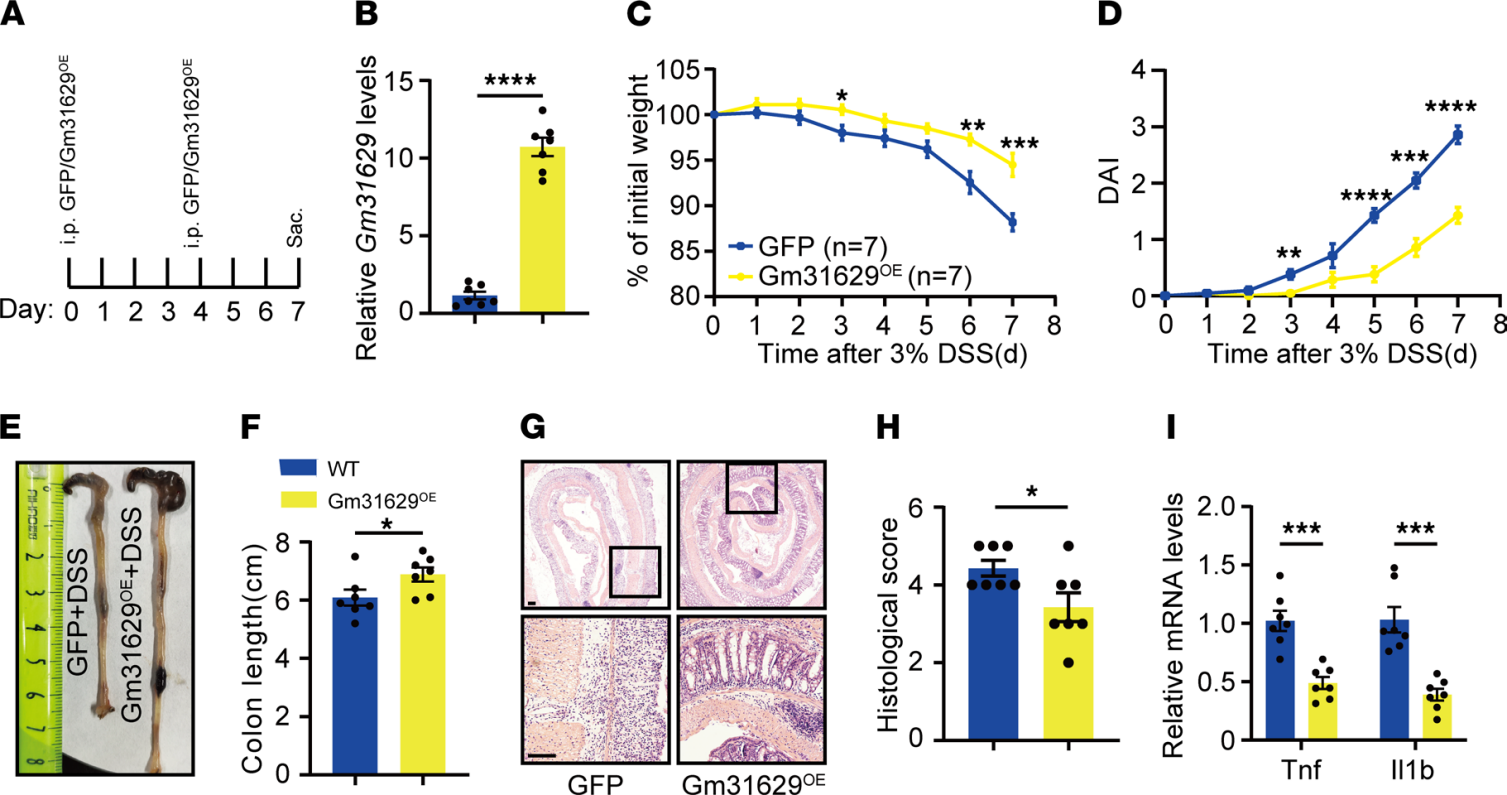
Overexpression of Gm31629 ameliorates DSS-induced colitis. Eight-week-old WT mice were i.p. injected with adenovirus overexpressing Gm31629 (Gm31629^OE^) and GFP (on day 0 and day 4 of DSS treatment) followed by 7-day 3% DSS treatment (*n* = 7). (**A**) Time schedule of adenovirus injection during DSS treatment. (**B**) qPCR analysis of *Gm31629* in colon tissues. (**C**) Body weight changes of mice. (**D**) DAI of mice during the indicated time. (**E**) Gross pictures of the colons. (**F**) Measurement of colon length in **E**. (**G**) H&E-stained section of colon tissues. Scale bar: 100 μm. (**H**) Quantification of histological score from colonic sections in **G**. (**I**) qPCR analysis of *Tnf* and *Il1b* in colon tissues. All data were represented as mean ± SEM. For comparisons of 2 groups, a 2-tailed Student’s *t* test was performed. *P < 0.05; **P < 0.01; ***P < 0.001; ****P < 0.0001.

Given that Gm31629 plays an important role in intestinal epithelial regeneration, we next tested whether Gm31629 overexpression in vivo could protect against DSS-induced colitis. Recombinant adenovirus was i.p. injected into the abdominal cavity twice to deliver Gm31629 on day 0 and day 4 of 7-day DSS treatment (Figure 6A). Overexpression of Gm31629 in colon tissues was confirmed by qPCR (Figure 6B). Gm31629-overexpressed mice showed reduced weight loss and scored lower DAI scores compared with control mice (Figure 6, C and D). Moreover, Gm31629-overexpressed mice preserved longer colon length (Figure 6, E and F). Histological evaluation based on H&E-stained sections confirmed attenuated inflammation and enhanced regeneration in Gm31629-overexpressed mice (Figure 6, G and H). Accordingly, mRNA levels of colonic *Tnf* and *Il1b* were decreased in Gm31629-overexpressed mice (Figure 6I). Therefore, overexpression of Gm31629 could help reduce DSS-induced inflammation and promote intestinal epithelial regeneration.

### TF2A mimics Gm31629 and ameliorates DSS-induced colitis.

**Figure 7:**
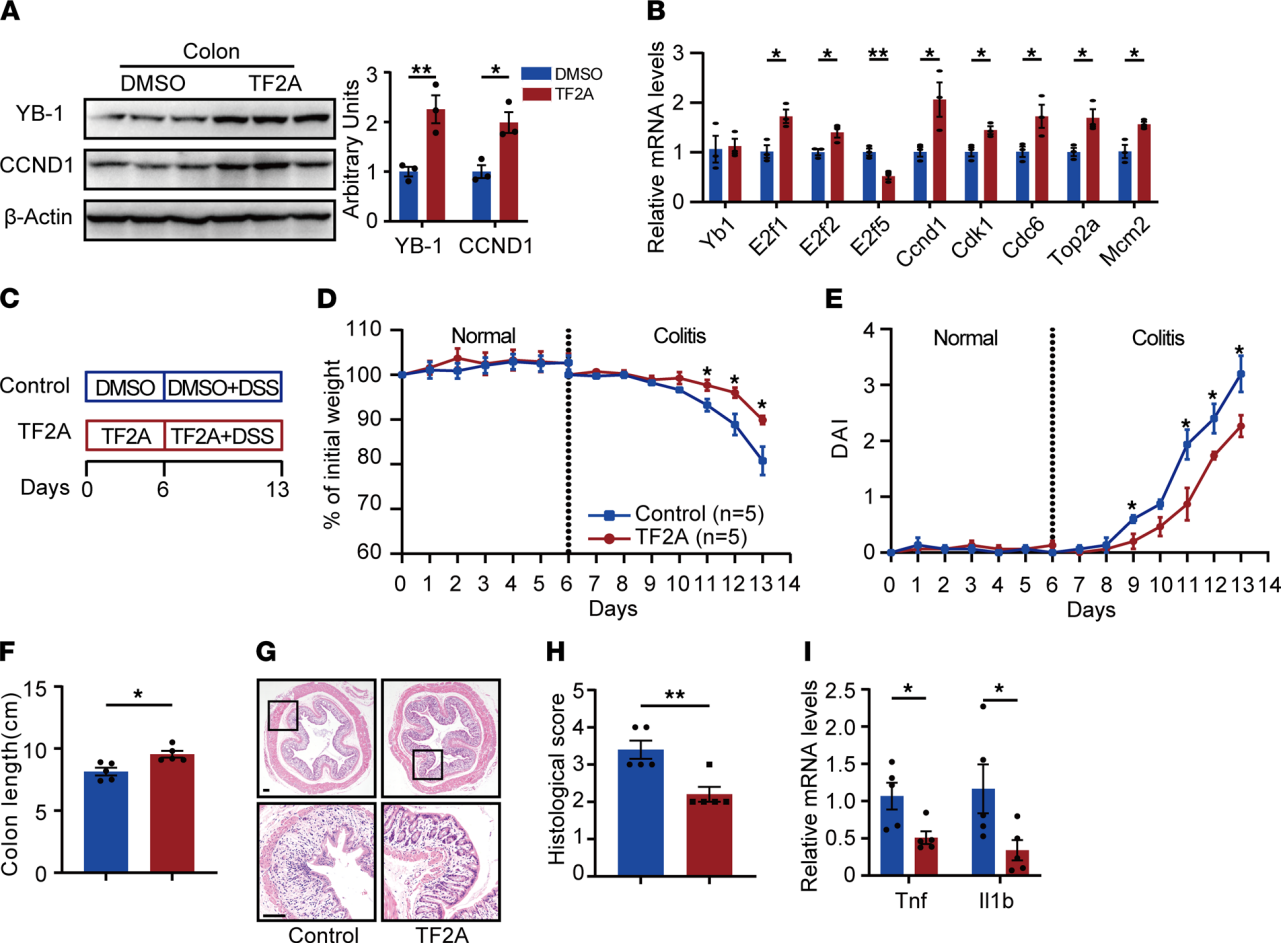
TF2A mimics Gm31629 and ameliorates DSS-induced colitis. (**A** and **B**) Eight-week-old WT mice were treated with TF2A for 7 days (*n* = 3). (**A**) The protein level of YB-1 and CCN1 in colon tissues. (**B**) qPCR analysis of *Yb-1* and E2F target genes in colon tissues. (**C**–**I**) Eight-week-old WT mice were subjected to TF2A treatment for 6 days, followed by TF2A plus 3% DSS treatment for 7 days, and sacrificed on day 14 for colitis monitor (*n* = 6). (**C**) Time schedule of TF2A and DSS treatment. (**D**) Body weight changes during normal and colitis state. (**E**) DAI of mice. (**F**) Measurement of colon length. (**G**) H&E-stained sections of colon tissues. Scale bar: 100 μm. (**H**) Quantification of histological score from colonic sections in **G**. (**I**) qPCR analysis of *Tnf* and *Il1b* of in colon tissues. All data were represented as mean ± SEM. For comparisons of 2 groups, a 2-tailed Student’s *t* test was performed. *P < 0.05; **P < 0.01.

We further suspected if small molecular compounds mimicking Gm31629 could ameliorate colitis. Our previous study validated the efficiency of a naturally occurring small compound, TF2A, on stabilizing YB-1 protein as a mimic of Gm31629 (14). We next tested the availability of TF2A in intestinal mucosal. Eight-week-old mice were administered TF2A and vehicle for 7 days under normal state. TF2A administration increased protein levels of YB-1 and CCND1 and mRNA levels of E2F target genes in colon tissues, indicating a potential role of TF2A in alleviating colitis (Figure 7, A and B). We further confirmed the effectiveness of TF2A under the colitis state. Mice at 8 weeks old were subjected to TF2A or vehicle treatment for 7 days prior to DSS administration (Figure 7C). Despite no apparent weight change or disease severity between 2 groups being observed under the normal state (Figure 7, D and E), TF2A treatment notably alleviated weight loss and lowered DAI scores under the colitis state (Figure 7, D and E). By the end of DSS treatment, the TF2A group preserved longer average colon length and scored lower histological scores (Figure 7, F–H). qPCR analysis further confirmed decreased colonic inflammation after TF2A treatment (Figure 7I). Taken together, the Gm31629 mimic TF2A might be a potential natural compound against colitis.

## Discussion

Inflammatory bowel disease is characterized by immune dysregulation and mucosal destruction (1, 3). Despite acquired advances in immunoregulation therapy, improving therapeutic outcomes without lethal side effects remains challenging (28). In this regard, new therapeutic approaches targeting intestinal epithelial regeneration are promising. In the present study, we found that the expression of Gm31629 in intestinal epithelium cells decreased in response to inflammatory stimulation, both in mice and humans. Remarkably, loss of Gm31629 led to exacerbated intestinal injury and delayed epithelial regeneration after DSS treatment. By contrast, genetic or pharmacological approaches to enhance Gm31629 expression protected against DSS-induced mucosal damage. These findings represent an important role of Gm31629 in intestinal epithelial regeneration.

lncRNAs have been reported to interact with nucleic acids and proteins, thus regulating downstream signaling (9, 10). In the present study, we demonstrate that Gm31629 promotes E2F pathways and cell proliferation by stabilizing YB-1 protein, thus facilitating epithelial regeneration. Mechanistically, Gm31629 can bind to YB-1 through molecular docking and protects it from ubiquitination-mediated degradation (14). Since lncRNAs can regulate gene expression levels at various levels such as epigenetic regulation, transcriptional regulation, and posttranscriptional regulation, it is possible that, in addition to YB-1, Gm31629 may interact with other potential targets to modulate epithelial regeneration. Besides, we noticed that not only the protein level of YB-1, but also the mRNA level of YB-1 decreased after DSS administration (Supplemental Figure 2, F and G). This result suggests that, in addition to Gm31629, other factors may regulate colonic YB-1 expression, though this awaits further investigation.

Gm31629 was widely expressed in the intestinal epithelium, which is composed of heterogenous cell types including intestinal stem cells, goblet cells, and intraepithelial lymphocytes. The gain and loss of function of Gm31629 in these cells might also regulate epithelial regeneration in response to inflammatory stimulation. Therefore, we could not exclude the possibility that other cell types in the epithelium may exert an indirect role on epithelial stem cell proliferation. However, we have shown in vitro in CT26.WT that Gm31629 deficiency induced cell cycle arrest in the S-phase and inhibited intestinal cell proliferation (Figure 4, A and B). Moreover, IHC results reflect that Ki-67, β-catenin, and LGR5 expression were significantly downregulated in the crypt, where intestinal stem cells mainly reside (Figure 4C). To examine the role of Gm31629 in regulation of intestinal regeneration in vivo, we performed a BrdU-labeling assay to detect proliferative activity in the LPS-challenged model. The results show significantly decreased BrdU^+^ cells in Gm31629^–/–^ mice (Figure 4, F and G). Therefore, we have concluded that Gm31629 deficiency affected colitis, at least in part, via directly regulating epithelial cell proliferation.

An umbrella review of meta-analyses has demonstrated that green tea consumption prevented ulcerative colitis (29); however, the benefits of nongreen tea and the underlying mechanism are unknown. TF2A is the major extract of black tea, in addition to theaflavin, theaflavin-3’-gallate, and theaflavin-3,3’-digallate (30). Ukil et al. showed that theaflavin-3,3’-digallate protected against TNBS-induced experimental colitis (31). A previous study also suggested the antiinflammatory effects of the mixture of TF2A and theaflavin-3’-gallate (32). Here, we demonstrate the protective effects of TF2A in experimental colitis on the basis of our previous study (14). Oral administration of TF2A restored protein level of YB-1 and, thereby, upregulated E2F target genes in colon tissues. Compared with the control group, TF2A treatment protected against DSS-induced inflammation and mucosal damage in vivo. These findings provide possible mechanistic bases for the protective effects of tea consumption and therapeutic approaches against colitis.

Taken together, lncRNA Gm31629 links intestinal inflammation and epithelial regeneration under inflammatory conditions, which provides a therapeutic clue for inflammatory bowel disease.

## Methods

### Animal models.

C57BL/6J mice were purchased from Hunan SJA Laboratory Animal Company. Gm31629^–/–^ mice were generated by TetraOne technique as previously reported (14, 22). Briefly, the 4.92 kb sequence of Gm31629 on Chromosome 1 was targeted as the KO region (Supplemental Figure 1B). Genotyping was processed by PCR using the following primers: forward: 5′-GCTAAGCTCGAGGGACCTAATA-3′, 5′-CTGCTGCTCACCTCTAGTCATT-3′, and reverse, 5′-AGGGATGTTCCTCACTGGCTGG-3′ (Supplemental Figure 1C). All mice were kept on the C57BL/6J background and housed in the specific pathogen–free animal facility (22-24°C) of Laboratory Animal Research Center of Central South University on a 12-hour dark/light cycle with ad libitum access to water and a regular chow diet. Mice were treated with 3% w/v DSS(36,000–50,000 molecular weight, MP Biochemicals) in drinking water for 7 days to induce acute ulcerative colitis (33). To examine colonic epithelial regeneration in vivo, mice were treated with 3.5% w/v DSS in drinking water for 5 days, and DSS was withdrawn for another 5 days (17). The severity of colitis was scored by the DAI according to body weight loss, presence of diarrhea, and presence of bloody stools as previously reported (33). For LPS challenge model, adult mice were treated with 2 mg/kg LPS (serotype O111:B4, MilliporeSigma) by i.p. injection for 24 hours (17). For therapeutic treatment, TF2A (Wako) was given by oral gavage at the dose of 10 mg/kg/day daily and was begun at 6 days before DSS administration (14). In this study, male mice at 8 weeks old were used.

### BrdU-labeling assay.

The method for a BrdU-labeling assay was modified from a previously described protocol (17). Briefly, mice were i.p. injected with 50 mg/kg BrdU (MilliporeSigma) at 18 hours after LPS challenge and sacrificed 24 hours after BrdU was given. The colons were collected for paraffin sections and anti-BrdU IHC staining. The number of Brdu^+^ proliferative epithelial cells was assessed.

### Clinical samples.

Human colon mucosal tissues from 13 patients with a clinical diagnosis of ulcerative colitis and active endoscopic inflammation were obtained through colonic biopsy conducted by the Department of Gastroenterology at Xiangya Hospital of Central South University.

### Recombinant adenovirus and in vivo gene delivery.

Purified adenovirus generated under the help of Obio Technology (Shanghai) Corp. Ltd. were i.p. injected for gene delivery (34). Virus vector pAdeno-EF1-Gm31629-MCMV-EGFP-3FLAG (Gm31629 OE) was applied for Gm31629 overexpression, and pAdeno-EF1-MCS-MCMV-EGFP-3FLAG (GFP) was applied as control. For each mouse, 1 × 10^9^ PFU of virus was injected i.p. (diluted in 100 μL PBS) on day 0 and day 4 of DSS treatment. Functional validation was verified by qPCR.

### Cell culture and treatment.

CT26.WT cells, provided by Procell Life Science & Technology Co. Ltd., were cultured in DMEM (Thermo Fisher Scientific) supplemented with 10% (v/v) FBS (Biological Industries) plus 50 U/mL penicillin and 50 μg/mL streptomycin (Solarbio) at 37°C in a humidified incubator with 5% CO_2_. Gm31629- or YB-1–targeted small interfering RNAs (siRNAs) were purchased. Transfection of siRNAs was performed using lipofectamine RNAiMAX (Invitrogen) following the instructions. qPCR or Western blot was used to determine the knockdown efficiency 2 days after transfection. For Gm31629 overexpression, adenovirus particles expressing Gm31629 or GFP were transfected by lipofectamine 2000 (Invitrogen) according to the manufacturer instructions.

### Cell proliferation assay.

Cell proliferation was measured using a CCK-8 kit (Dojindo) according to manufacture protocol. Briefly, CT26.WT cells were seeded into 96-well plates at a density of 3 × 10^3^ cells per well. After cells adhered to the plates, cells were transfected with Gm31629 targeted siRNA or control siRNA. A 10 μL CCK-8 solution was added to each well at 0, 12, 24, and 48 hours after transfection and incubated at 37°C for 1 hour before measuring the absorbance at 450 nm.

### Cell cycle analysis.

Cell cycle analysis was examined by flow cytometry. Briefly, 50%–60% confluent CT26.WT cells cultured in 12-well plates were serum starved for 6 hours before siRNA transfection to synchronize the cell cycle. The cells were harvested 48 hours after transfection and stained using a Cell Cycle Staining Kit (MultiSciences). Stained samples were tested by flow cytometry to detect the DNA content, and cell cycle analysis was processed by FlowJo software (FlowJo LLC).

### RNA-Seq and bioinformatics analysis.

Total RNAs pooled from colon tissues of 3 WT mice and 3 Gm31629^–/–^ mice treated with 3% DSS were subjected to commercial RNA-Seq (Annoroad Gene Technology). Differentially expressed genes were identified by DEGseq on the basis of *P* < 0.05 and |log_2_ fold change| > 1. Heatmaps were generated to show gene expression differences in different groups using morpheus (https://software.broadinstitute.org/morpheus). GO enrichment was performed using KOBAS (http://kobas.cbi.pku.edu.cn/) to show the biological processes affected among different groups. For GSEA, all protein-coding genes were token, except for those with low expression (read counts < 1). Selected genes were mapped to human orthologue genes by metascape and loaded into GSEA to identify enriched hallmarks gene sets (MSigDBv7.0) using default parameters. The accession nos. for the RNA-Seq data reported in this report are available in GEO database (GSE168612 https://www.ncbi.nlm.nih.gov/geo/query/acc.cgi?acc=GSE168612 and GSE137869 https://www.ncbi.nlm.nih.gov/geo/query/acc.cgi?acc=GSE137869).

### qPCR.

Total RNAs were extracted from tissue or cultured cells using the E.Z.N.A Total RNA Kit (OMEGA). Afterward, single-strand cDNA synthesis and qPCR analysis were performed as previously reported (35). The primer pairs used for qPCR were listed in Supplemental Table 1. The relative mRNA levels of target genes were given by the 2^−ΔΔCt^ method using β-actin as an internal control.

### ELISA.

Serum TNF-α and IL-1β protein levels were quantified using ELISA kits according to manufacturer’s instructions (Nanjing Jiancheng Bioengineering Institute, Nanjing, China).

### Histochemistry and IHC.

The mouse colon samples were fixed in 4% paraformaldehyde for 24 hours and embedded in paraffin. For inflammation-associated histological measurement, sections were stained with H&E and graded in terms of tissue damage and inflammation cell infiltration from 0 to 3 as previously described (33). The IHC staining was processed as previously reported (36). The primary antibodies used in IHC staining are: mouse anti–Ki-67 (Proteintech, 27309-1-AP), rabbit anti–β-catenin (Cell Signaling Technology, 9562), mouse anti-Lgr5 (Origene, TA503316), and mouse anti-BrdU (Cell Signaling Technology, 5293).

### FISH.

FISH was performed using a Fluorescence in situ Hybridization Kit (Ribobio, C10910). Gm31629 targeted FISH probe (Ribobio, lnc1100239) was purchased from Ribobio. The probes were detected with red fluorescence of Cy3. Briefly, mouse colon samples were fixed in 4% paraformaldehyde, dehydrated in 20% and 30% sucrose, and embedded in OCT. Frozen 8 μm–thick sections were incubated with Gm31629-targeted probes according to the manufacture’s protocol. The nuclei were counterstained with DAPI/Antifade (Vectashield) and observed using a fluorescence microscope system.

### Western blot analysis.

Western blot analysis was performed as previously reported (37). The primary antibodies used in Western blot analysis are: rabbit anti–YB-1 (Abcam, ab12148), mouse anti-CCND1 (Proteintech, 60186-1-Ig), mouse anti–β-actin (Proteintech, 66009-1-Ig), rabbit anti–β-catenin (Cell Signaling Technology, 9562), mouse anti-Lgr5 (Origene, TA503316), and mouse anti-PCNA (Boster, BM0104).

### Statistics.

All data were presented as the means ± SEM from at least 4 animals or 3 independent experiments, and representative results are shown. For comparisons of 2 groups, a 2-tailed Student’s *t* test was performed. Differences were considered significant at *P* value less than 0.05 (*P < 0.05; **P < 0.01; ***P < 0.001; ****P < 0.0001).

### Study approval.

All animal experimental procedures and protocols were reviewed and approved by the Animal Care and Use Committees of the Laboratory Animal Research Center at Xiangya Hospital of Central South University. For clinical samples collection, the study was approved by the Ethics Committee at Xiangya Hospital of Central South University, and written informed consent was obtained from all participants prior to colonic biopsy.

## Author contributions

HYZ, XWL, XHL, and XF designed the experiments. XF carried out most of the experiments, generated data, and drafted the manuscript. YX, JH, MY, QG, TS, YH, JY, and CJL helped to collect the samples. XHL is the guarantor of this work. HYZ, XHL, and XHL proofread and revised the manuscript. HYZ supervised the experiments, analyzed results, and takes the responsibility for data accuracy. All authors had access to the study data and had reviewed and approved the final manuscript.

## Supplementary Material

Supplemental data

## Version 1 02/10/2022

In-Press Preview

## Version 2 03/22/2022

Electronic publication
