# Supplementary material for: Long noncoding RNA Gm31629 protects against mucosal damage in experimental colitis via YB-1/E2F pathway
Source: JCI Insight. 2022 Mar 22;7(6):e150091. doi: 10.1172/jci.insight.150091 (PMC8986069; doi:10.1172/jci.insight.150091)
Supplement: Supplemental data [file jciinsight-7-150091-s181.pdf]

Supplementary Table 1

Nucleotide sequences of primers used for qRT-PCR

Mouse	F: GATCATTGCTCCTCCTGAGC
Actin	R: ACTCCTGCTTGCTGATCCAC
mouse	F: CTAAGCGAACTCGGGAGC
Gm31629	R: CACAGCAGGATTGATGGATG
Human	F: CGTGGACATCCGCAAAGAC
Actin	R: TCGTCATACTCCTGCTTGCTG
Human	F: GGAGTCCAGAAGGCGGTAGT
Gm31629	R: CTGAAAGTAGGTGCCGAGGA
Mouse	F: CCCTCACACTCAGATCATCTTCT
Tnf	R: GCTACGACGTGGGCTACAG
Mouse	F: GCAACTGTTCCTGAACTCAACT
Il1b	R: ATCTTTTGGGGTCCGTCAACT
Mouse	F: CAGACCGTAACCATTATAGACGC
Yb-1	R: ATCCCTCGTTCTTTTCCCCAC
Mouse	F: TGCAGAAACGGCGCATCTAT
E2f1	R: CCGCTTACCAATCCCCACC
Mouse	F: ACGGCGCAACCTACAAAGAG
E2f2	R: GTCTGCGTGTAAAGCGAAGT

Mouse	F: ACTCAGGGCCTATCCATGTG
E2f5	R: CTCAGGCAGGTTTTGAGCAG
Mouse	F: GCGTACCCTGACACCAATCTC
Ccnd1	R: CTCCTCTTCGCACTTCTGCTC
Mouse	F: AGAAGGTACTTACGGTGTGGT
Cdk1	R: GAGAGATTTCCCGAATTGCAGT
Mouse	F: TGGCATCATACAAGTTTGTGTGG
Cdc6	R: CAGGCTGGACGTTTCTAAGTTTT
Mouse	F: CAACTGGAACATATACTGCTCCG
Top2a	R: GGGTCCCTTTGTTTGTTATCAGC
Mouse	F: ATCCACCACCGCTTCAAGAAC
Mcm2	R: TACCACCAAACTCTCACGGTT
Mouse	F: CCTACTCGAAGACTTACCCAGT
Lgr5	R: GCATTGGGGTGAATGATAGCA
Mouse	F: AAGCACACCTTGACTGGTACG
Ascl2	R: AAGTGGACGTTTGCACCTTCA

(F, forward primer; R, reverse primer.)

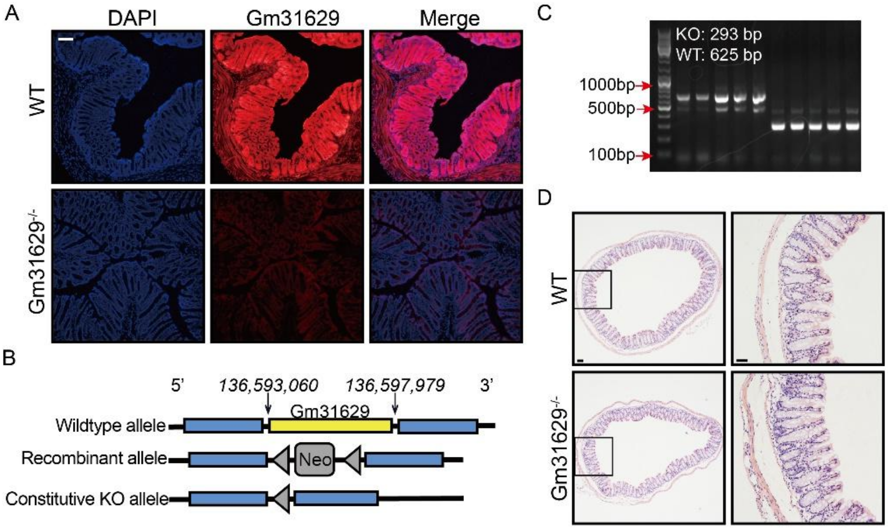

Supplementary Figure 1. Construction of Gm31629 knockout mouse

(A) Representative fluorescence in situ hybridization image of Gm31629 (Red) in Gm31629$^{-/-}$ mice and WT mice (Scale Bar 100μm). (B)Scheme of the mouse Gm31629 knockout construct. (C) PCR image of genotyping data of Gm31629$^{-/-}$ mice and WT mice. (D) H&E stained sections of colon tissues (Scale Bar 100μm)

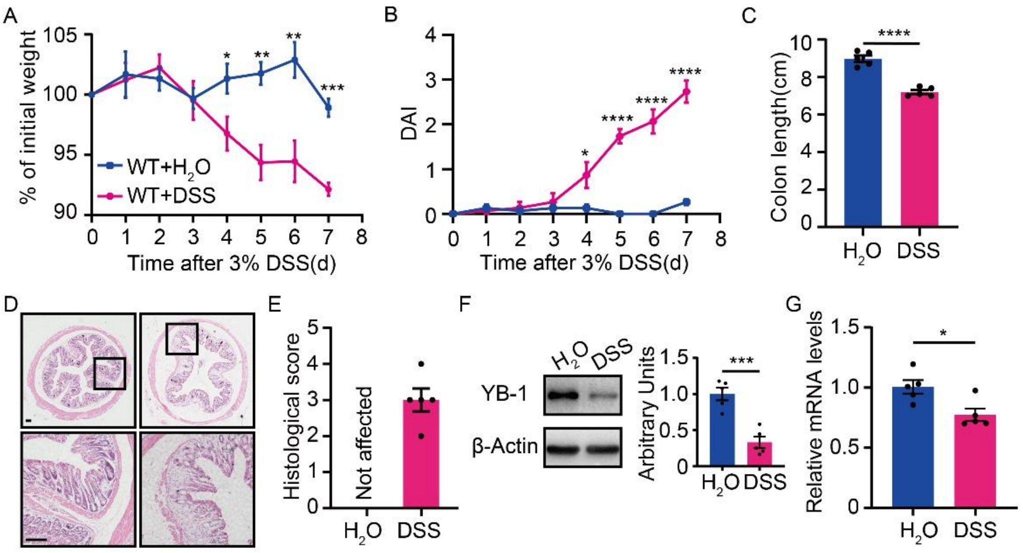

Supplementary Figure 2. Intestinal YB-1 decreases in response to inflammatory stimuli

(A-E) Eight-week-old WT mice were subjected to 3%DSS in drinking water or tap water for 7 days and sacrificed on day 8 (n=5). (A) Body weight changes of mice. (B) Disease activity index (DAI) of mice during indicated period. (C) Measurement of colon length. (D) H&E stained sections of colon tissues (Scale Bar 100μm) (E) Quantification of histological score from colonic sections in panel D (F) The protein level of YB-1 in colon tissues. (G) qRT-PCR analysis of *Yb-1* in colon tissues. All data were represented as mean ± SEM. For comparisons of two groups, a two-tailed Student's t test was performed.
